# Infections in Inborn Errors of Immunity with Combined Immune Deficiency: A Review

**DOI:** 10.3390/pathogens12020272

**Published:** 2023-02-07

**Authors:** Kalpana George, Geeta Govindaraj

**Affiliations:** 1 Department of Microbiology, Government Medical College, Kozhikode 673008, Kerala, India; 2 FPID Regional Diagnostic Centre, Government Medical College, Kozhikode 673008, Kerala, India; 3 School of Family Health Studies, Kerala University of Health Sciences, Kozhikode 673008, Kerala, India; 4 CSIR-IGIB, DHR, SERB, FPID Program on Primary Immunodeficiency Disorders, Kozhikode 673008, Kerala, India

**Keywords:** infections, inborn errors of immunity, combined immune deficiency

## Abstract

Enhanced susceptibility to microbes, often resulting in severe, intractable and frequent infections due to usually innocuous organisms at uncommon sites, is the most striking feature in individuals with an inborn error of immunity. In this narrative review, based on the International Union of Immunological Societies’ 2022 (IUIS 2022) Update on phenotypic classification of human inborn errors of immunity, the focus is on commonly encountered Combined Immunodeficiency Disorders (CIDs) with susceptibility to infections. Combined immune deficiency disorders are usually commensurate with survival beyond infancy unlike Severe Combined Immune Deficiency (SCID) and are often associated with clinical features of a syndromic nature. Defective humoral and cellular immune responses result in susceptibility to a broad range of microbial infections. Although disease onset is usually in early childhood, mild defects may present in late childhood or even in adulthood. A precise diagnosis is imperative not only for determining management strategies, but also for providing accurate genetic counseling, including prenatal diagnosis, and also in deciding empiric treatment of infections upfront before investigation reports are available.

## 1. Introduction

Inborn errors of immunity comprise a plethora of single gene defects, resulting in the impaired structure or functioning of the immune system, that are rare individually, but account for a significant burden of morbidity and mortality when considered together. Our understanding of basic and clinical immunology and molecular medicine has benefited immensely from the application of next generation sequencing technology to the study of inborn errors of immunity, of which 485 distinct disorders have been described so far and this review is based on the International Union of Immunological Societies 2022 Update [1]. Increased susceptibility to a range of pathogens or to a single pathogen resulting in severe, recurrent, intractable or unusual infections is the most common presentation of an inborn error of immunity. The SARS-CoV2 pandemic has resulted in the delineation of genetic defects and molecular mechanisms that underlie critical COVID-19 in a subset of patients, and has served to galvanize research into inborn errors of immunity [2]. In this narrative review, the focus is on commonly encountered combined inborn errors of immunity with susceptibility to infections.

Combined immune deficiency disorders that involve multiple components of the immune system result in susceptibility to a broad range of pathogens due to defective adaptive and cellular immunological processes. Unlike Severe Combined Immune Deficiency, these disorders are commensurate with survival beyond infancy and several of these disorders are associated with clinical characteristics that are of a syndromic nature. Although combined immune deficiency disorders often present with infections in the first two years of life, milder defects may present later in childhood or even in adults with features of autoimmunity, immune dysregulation, autoinflammation or malignancy [3,4,5]. Mutations in a particular gene may result in variable severities of immune dysfunction depending on the degree of penetrance and on the resulting functional defect [6].

Recurrent or persistent sinopulmonary infections, infections with opportunistic pathogens, failure to thrive, chronic diarrhoea, lymphoproliferation associated with EBV infection, recent onset autoimmunity, persistent lymphopenia and a family history suggestive of an inborn error of immunity warrant evaluation for an inherited combined immune deficiency. Apart from these, new onset autoimmune conditions or lymphopenia and unexplained weight loss may be the presentations in adulthood.

The spectrum of infections in the different inborn errors of immunity with combined immune deficiency is represented in Table 1.

## 2. Spectrum of Infections

### 2.1. Recurrent Sinopulmonary Infections

Recurrent sinopulmonary infections are by far the commonest presentation of an underlying inborn error of immunity. Increased exposure to pathogens as occurs at school entry or when a sibling starts going to school is also commonly associated with an increase in the number of sinopulmonary infections. However, these episodes are usually viral upper respiratory infections and are self-limiting. Anatomical defects including trachea—oesophageal fistula, sequestration and foreign body in the bronchial tree lead to sinopulmonary infections that are localized. Upper respiratory infections like rhinosinusitis may also result from an underlying inborn error of immunity, but are often associated with infections at other sites including otitis and pneumonia.

Pneumonia which is recurrent, especially with intermittent radiological clearing of lesions [85], or is associated with complications like lung abscesses, pneumatoceles or empyema [86], or caused due to unusual pathogens is a hallmark of an inborn error of immunity. Reasons for a secondary immunocompromised state or ciliary dysfunction should be ruled out upfront.

Recurrent sinopulmonary infections in combined immune deficiency disorders are caused by a broad range of pathogens including viruses and opportunistic pathogens. Opportunistic pathogens often cause recurrent respiratory infections in CD40 ligand/CD 40 deficiency and include PCP, CMV, Histoplasma and *Cryptococcus* sp. [87,88,89], but are unusual in patients with Ataxia Telangiectasia [53].

Bronchiectasis, a chronic suppurative lung disease, occurs following recurrent pyogenic infections in autosomal dominant Hyper IgE syndrome, ataxia telangiectasia and activating mutations of phosphoinositide 3-kinase delta (PI3KD) syndrome [90].

### 2.2. Gastrointestinal Infections

Gastrointestinal tract is largest organ and is constantly exposed to foreign antigens [91]. Gastrointestinal manifestations occur in 5–50% of patients with inborn errors of immunity [92] and may be the most prominent presenting feature in a subgroup of these patients with Combined Immunodeficiency [93]. Infections of the gastrointestinal tract resulting in diarrhea are the commonest manifestation and are usually more severe, last longer, require prolonged treatment and are caused by opportunistic pathogens. Infections in combined immunodeficiency disorders may be of bacterial, viral or fungal aetiology [94]. In MHC class 11 deficiency, gastrointestinal infections due to *Shigella*, *Salmonella*, *Staphylococcus aureus*, *Escherichia coli*, *Campylobacter,* adenovirus or enterovirus may occur [22] while candidal infections occur in patients with DiGeorge syndrome and Hyper IgE syndrome. Disseminated GI infections due to *Cryptococcus* and *Histoplasma* may occur in Hyper IgE syndrome [95,96].

### 2.3. Infections of the Central Nervous System

Infections of the central nervous system may result in mortality or severe morbidity among patients with an underlying Combined Immunodeficiency Disorder. Meningitis, meningoencephalitis, progressive multifocal leukoencephalopathy and chronic lymphocytic meningitis caused predominantly by viruses are frequently encountered. Bacteria, fungi and protozoan parasites can also infect the central nervous system in these patients.

Enteroviruses are frequently reported in patients suffering from Hyper IgM syndrome [97,98]. Cerebral toxoplasmosis has been reported in a middle aged male as the first presentation of CD40L deficiency [99]. JC virus infection can sometimes take a rapid downhill course [100]. Apart from Enteroviruses, *E.coli* can cause meningitis in MHC class II [31]. Recurrent Staphylococcal meningitis may be an indicator of Hyper IgE syndrome [101]. Anticapsular antibody deficiency is encountered in conditions like Wiskott Aldrich syndrome and Di George syndrome leading to increased risk of meningitis by capsulated bacteria [102,103].

### 2.4. Mucocutaneous Infections

Mucocutaneous infections that are persistent or refractory to treatment may be the first sign of underlying inborn errors of immunity including combined immune deficiency disorders.

Bacterial skin infections, especially furuncles and abscesses that lack signs of inflammation, the ‘cold abscesses’ are a hallmark of autosomal dominant loss of function Hyper IgE syndrome [68], while disseminated mucocutaneous viral infections like warts and molluscum contagiosum occur in autosomal recessive Hyper IgE syndrome due to *DOCK8* variants apart from eczema, abscesses and mucocutaneous candidiasis [41,42]. Mucocutaneous candidiasis is also commonly observed in patients with AD Hyper IgE syndrome, and median rhomboid glossitis has also been reported [104].

Persistent candidal infections of the skin, oral cavity, oesophagus and vagina may be the presenting feature of an inborn error of immunity especially associated with T cell defects [105], but HIV infection, diabetes and immunosuppressive therapy need to be excluded upfront. Defective IL - 17 responses have been found to underlie most cases of mucocutaneous candidiasis, but in immunocompetent individuals intact Th17 responses are protective against infection despite colonization of skin and other surfaces [106]. Identification of the underlying inborn error of immunity is important to decide on therapeutic options since azole resistance is not uncommon [107]. STAT1 gain of function mutations have been found to result in impaired STAT3 responses resulting in mucocutaneous candidiasis [108].

### 2.5. Opportunistic Infections

Opportunistic infections due to bacteria, viruses, fungi or commensals that do not usually cause infections in immunocompetent hosts, may lead to life threatening infections among individuals with combined immunodeficiency, and may be the first manifestation as well [109]. Most experience and published work on opportunistic infections is associated with HIV infection. The type of opportunistic infections may give a clue to the underlying inborn error of immunity [110].

Autosomal recessive Hyper IgE syndrome due to DOCK8 mutations is associated with generalized molluscum contagiosum, HPV, herpes simplex infections, risk of development of malignancy and /CNS vasculitis [41,42]. WHIM syndrome is associated with predisposition to HPV infections resulting in multiple warts of the hands, feet and trunk and also increased susceptibility to condyloma acuminata, varicella zoster and herpes simplex infections.

Candida sp. are the most common cause of invasive fungal disease in children [111], while Aspergillus is the commonest cause of invasive mold disease followed by *Mucorales* sp. [112] Opportunistic infections may also occur following the administration of live vaccines.

### 2.6. Infections following Immunization

It is important to consider the risk—benefit ratio when immunizing individuals with an inborn error of immunity, considering the impaired vaccine efficacy as well as the propensity of live vaccines to result in untoward adverse reactions that may even be life threatening.

Live vaccines result in adverse events due to disseminated infections amongst patients with combined immunodeficiency disorders including vaccine—associated paralytic polio, disseminated BCGiosis and chronic rotavirus infection [113]. However, live viral vaccines like measles, mumps, rubella and varicella vaccines have been found to be safe in children with partial DiGeorge syndrome [114]. It has been suggested that a life - threatening or fatal viral illness should result in comprehensive genetic evaluation even if the immunological investigations are normal [115]. Absence of newborn screening programs for severe inborn errors of immunity in much of the developing world is an impediment to prevention of adverse events following immunization in this group of children. A review of 121 case reports of disseminated BCG infection yielded 61 cases of an underlying immune deficiency disorder [116]. Adverse reactions following BCG vaccine most commonly result in lymphadenopathy involving the axillary, cervical, mediastinal or mesenteric lymph nodes and are usually mild [117]. Disseminated BCG reactions in children warrant a search for an underlying inborn error of immunity, since they are often the first manifestation that comes to medical attention [118].

## 3. Preventive Measures

Impeccable attention to environmental and personal hygiene, prevention of contact with sick individuals including avoidance of unnecessary hospital visits, antimicrobial prophylaxis and immunoglobulin replacement when indicated are the cornerstones of prevention of infection among individuals with inborn errors of immunity, including those with combined immune deficiency. Use of irradiated blood and blood products and immunization with vaccines likely to afford protection without resulting in disseminated infection are other preventive strategies. However, curative treatment modalities like hematopoietic stem cell transplants or gene therapy are necessary since all infections are not preventable with antimicrobial and/or immunoglobulin prophylaxis.

In children with severe defects of cell mediated immunity, all live vaccines are contraindicated and killed vaccines are not likely to be efficacious. However, in case of milder defects like partial DiGeorge syndrome, live vaccines may be administered if CD3 counts are above 500 cells/ mm^3^ [114]. Human Papillomavirus vaccine is especially indicated in all IEIs with increased susceptibility to HPV including DOCK8 deficiency, Ataxia Telangiectasia, Netherton syndrome, Wiskott Aldrich syndrome STK4 and CD40 ligand deficiency [113].

It is important to ensure that family contacts are also protected from infections by being immunized with killed vaccines like influenza vaccine, and do not receive live vaccines likely to result in severe infections in immunocompromised children including oral polio vaccine and live influenza vaccine.(2011 National centre for Immunization and Respiratory Diseases) Measles, mumps, rubella vaccine and varicella vaccine may be given to household contacts, who however should be isolated if they develop a rash. In chronic pulmonary diseases, attention to chest physiotherapy and postural drainage are also priorities apart from antimicrobial prophylaxis and bronchodilators where indicated.

Commercial immunoglobulin preparations do not usually contain neutralizing antibodies to SARS-CoV-2 [119] and hence, all patients with combined immunodeficiency should receive SARS-CoV-2 vaccine, apart from continuing to use protective measures like masking. No serious adverse effects have been reported among patients who have received SARS-CoV-2 vaccine and studies on development of protective immunity have showed encouraging results [120,121,122].

## 4. Combined Immunodeficiency (CID), Generally Less Profound than SCID

### 4.1. CD40L and CD40 Deficiency

Maturation of antibody response following an encounter with antigen is marked by two events: class switch recombination (CSR) and somatic hypermutation (SHM). Interactions between activated CD4 T cells transiently expressing CD40L and B cells expressing CD40 provide key signals for B cells to generate proteins/enzymes resulting in CSR and SHM. Inherited defects in the CD40-CD40L axis result in hyper IgM (HIGM) syndrome characterised by normal to increased IgM and markedly reduced serum concentrations of IgG, IgA and IgE owing to faulty CSR and SHM [10]. CD40L deficiency has an X linked inheritance whereas CD40 is inherited in an autosomal recessive manner. Both conditions can result in a clinical picture of combined immunodeficiency and are indistinguishable clinically [12,123].

Patients with HIGM resulting from mutations in CD40-CD40L present during childhood itself with recurrent bacterial, viral and opportunistic infections. More than 80% of affected present with either upper or lower respiratory tract infections. In a case series of 56 patients, Pneumocystis jirovecii was isolated in 18 out of 22 patients who had interstitial pneumonia [55]. Other agents like CMV, Adenovirus, RSV, HSV type 1, *Pseudomonas*, *Pneumococcus*, *Staphylococcus, Cryptococcus neoformans, Mycobacterium bovi*s and atypical *Mycobacterium* can also cause infections [9,11,124,125]. In a cohort of 40 Chinese patients diagnosed with CD 40L deficiency, 6 patients presented with upper respiratory tract infection and later on *Talaromyces marneffei* was isolated from blood or bone marrow [8].

Gastrointestinal manifestations like recurrent and protracted diarrhoea are seen in approximately 50% of patients in various studies [8,9,126]. *Cryptosporidium parvum* causing protracted diarrhoea is a classical feature in HIGM. Other agents like *Clostridium difficile, Giardia lamblia, Campylobacter jejuni, Salmonella* sp., *Entamoeba histolytica, Isospora beli, Microsporidium* sp., and rotavirus have also been reported [127,128,129]. In approximately 50% of patients, it may not be possible to identify an etiological agent for protracted diarrhoea [11]. Hepatitis C virus, ECHO virus, histoplasmosis and Bartonella can cause hepatitis whereas *Cryptosporidium* is a major pathogen resulting in sclerosing cholangitis in this group of patients [9,11].

Central nervous system infections are seen in less than 20% of CD40L deficient patients. Agents like *Toxoplasma gondii*, ECHO virus, *Cryptococcus neoformans*, JC virus, CMV, *Streptococcus pneumoniae* and *Mycobacterium bovis* have been isolated from these patients [9,10,99].

### 4.2. Deficient Expression of MHC I & II

MHC Class I & II molecules have a major role in mounting normal immune responses by presenting processed antigens to CD4 and CD8 cells. MHC class I molecules are expressed ubiquitously on all nucleated cells and platelets whereas expression of MHC class II molecules is restricted to the surface of cells of the immune system [20]. In general, deficient expression of MHC Class I and II is known as ‘Bare lymphocyte syndrome’ (BLS) which is divided into Type I, Type II and Type III based on the MHC which is defective [130].

#### 4.2.1. Type 1 BLS

Patients with defective surface expression of MHC Class I molecule but intact class II expression are grouped under type 1 [18]. This is an extremely rare autosomal recessive condition. Type 1 BLS has been grouped into three based on clinical and immunological features.

Group 1: Patients in this group have a markedly reduced expression of MHC Class I and β2 microglobulin. In this less well elucidated group of patients, symptoms start as early as 4–5 months with recurrent bacterial, parasitic and fungal infections. Death occurs within the first three years of life [17,130].

Group 2: These patients have approximately a 10 fold reduction in surface expression of MHC Class I and β2 microglobulin and they are by and large asymptomatic [14,16].

Group 3: This is the best characterised phenotype among type 1 BLS resulting from defective genes coding for transporter associated with antigen processing proteins subunits TAP1/TAP2 and tapasin which are essential for cytosolic transport and loading of processed peptides onto HLA class I molecules before antigen presentation [16,17,18,130]. The patients usually present within the first six years of life with recurrent bacterial infections of the upper respiratory tract which includes rhinitis, sinusitis and otitis media. During the second decade of life lower respiratory tract involvement becomes more prominent. Multiple attacks of bronchitis and bacterial pneumonia eventually lead to bronchiectasis [16,17,18]. Commonly isolated bacteria include *Haemophilus influenzae, Streptococcus pneumoniae, Staphylococcus aureus, Klebsiella species, Escherichia coli* and *Pseudomonas aeruginosa* [17]. Surprisingly, immunity to a number viruses are substantially preserved. This is probably due to the existence of TAP independent mechanisms against viral invasion [130,131].

#### 4.2.2. Type 2 BLS

This is an autosomal recessive condition resulting in a defect in expression of class II MHC, either constitutional or inducible, and is grouped under type 2 BLS. Approximately 200 patients are reported worldwide. Majority of patients are from North Africa and the rest from mixed ethnic origin [18]. This is the most well studied BLS and is a heterogenous group which has four distinct complementation groups. The disease is due to defects in regulatory factors controlling transcription of MHC class II genes. four regulatory genes -*CIITA, RFXANK, RFXAP* and *RFX5*-are shown to be mutated in these patients. Affected individuals usually present recurrent infections of gastrointestinal tract and respiratory system and septicaemia. Mean age at first infection is 4.5 months (range 2 to 12 months) [20,21]. Patients are susceptible to bacterial, viral, protozoal and fungal infections. In a series of 30 patients reported by Klein et al, it was found that the most frequently isolated bacteria were *Pseudomonas* species (15 patients), *Salmonella* species (7 patients), pathogenic *Escherichia coli* species (6 patients), *Streptococcus* (6 patients), *Staphylococcus* species (5 patients), *Haemophilus* and *Proteus* species have been isolated from three patients each. Twenty three patients had severe, persistent viral infections, the most common being CMV, enterovirus, adenovirus, and herpes simplex virus. Eight patients acquired invasive Candida infections. Recurrent gastrointestinal tract infection with protracted diarrhoea and malabsorption was seen in majority of patients. Apart from different bacteria, Candida, *Giardia lamblia* and *Cryptosporidium* were isolated from stools of these patients [23]. In a Tunisian series of 34 patients, protracted diarrhoea was present in 26 patients. Cryptosporidium, vaccinal Poliovirus and E.coli were isolated from the intestinal tract of several of these patients [36]. Aluri et al reported MHC class II deficiency in 5 patients from India. The predominant clinical manifestation was recurrent lower respiratory tract infection. Bloodstream infections with opportunistic pathogens like *Burkholderia cepacia* and *Chryseobacterium indologenes* were reported in one each of these patients [26]. These patients can develop disseminated BCG infection and acute flaccid paralysis following oral polio vaccination [29,30].

Chronic enterocolitis and protracted diarrhoea caused by bacteria like Pseudomonas aeruginosa, Escherichia coli, Salmonella enteritidis, Klebsiella pneumoniae, Enterobacter cloacae, Campylobacter jejuni, Proteus mirabilis, and P. morgani, opportunistic fungi like Candida, protozoan parasites like G.lamblia and C. parvum has been reported from around the globe [19,20,21,22,23,24,25,31,32,34,35,36]. Sclerosing cholangitis has been reported in 10% of patients with MHC class II deficiency and Cryptosporidium parvum infection was reported in few of these patients. In Klein’s series three out of four patients who had sclerosing cholangitis had cryptosporidial infection. [20,21,22,23,36]. Bacterial cholangitis has also been reported associated with Pseudomonas, Enterococcus and Streptococcus Spp infections [21,23].

Recurrent pneumonia by Streptococcus pneumoniae, Staphylococcus aureus, Haemophilus influenzae and Pneumocystis jirovecii have been reported by many workers [21,23,27,35]. Meningoencephalitis and diffuse interstitial pneumonia by viruses can cause life threatening infections in these patients [20,25,29,31,32].

#### 4.2.3. Type 3 BLS

This type of BLS is identified by the absence of both MHC Class I and II molecules. These patients are severely immunosuppressed and survive only for a few years. Presentation is similar to Type 1 and 2 BLS [130].

### 4.3. ICOS Deficiency

Inducible co-stimulator (ICOS) is a T cell receptor that plays a major role in germinal centre formation, B cell differentiation and effector T cell responses. ICOS deficiency results in CVID-like disease [13]. Affected individuals have impaired formation of memory B cells and defective switched antibody responses. Peripheral B cell counts are decreased in adults whereas children at the time of diagnosis have a normal number of B cells. Naive, memory and effector T cell distribution can be normal. Some patients may have an inverted CD4/CD8 ratio. However circulating CXCR5+ CD4+ follicular T-helper-cell numbers are reduced in all patients. NK cell lymphopenia is also observed in a fraction of patients. The average age of presentation is 14 years ranging from 1 month to 39 years [13,132]. As in other CVID, recurrent respiratory tract infections are the most common cause of clinical presentation [133]. Infections with various encapsulated bacteria, viruses like *Herpes simplex,* HHV 6 and CMV, opportunistic fungal and parasitic agents have been reported. Respiratory failure due to *Pneumocystis jirovecii* has been reported in a 2 year old patient [134]. Gastrointestinal infections with *Giardia lamblia* and *Salmonella spp*. has also been noted [132,133]. Other agents associated with intestinal infections are *Campylobacter*, norovirus, adenovirus *Cryptosporidium parvum.* One patient developed squamous epidermal carcinoma of vulva associated with Human papilloma virus at the age of 34 years [13].

### 4.4. DOCK8 Deficiency

Mutations or deletions in the dedicator of cytokinesis 8 (*DOCK8*) are responsible for autosomal recessive variants of hyper IgE syndrome (HIES) resulting in combined immunodeficiency with severe atopy. Typical skeletal abnormalities seen in autosomal dominant HIES are absent in these patients [135]. The characteristic neonatal rash and facial dysmorphism are also not observed. It is believed that DOCK8 is essential for cytoskeletal rearrangements required for T cell activation and effector functions. There is defective activation of Th17 cells. Immunological assessment shows lymphopenia, where most of the patients have decreased CD4 and CD8 cells,B lymphocytes and NK cells. There is reduced NK cell [136] and CD8 and regulatory T cell function [137]. Increased absolute eosinophil counts are seen in almost all patients. Elevated IgE and declining IgM are the other prominent laboratory findings [41]. Affected patients have a predilection for treatment resistant viral skin infections especially, molluscum contagiosum, herpes zoster and recurrent herpes simplex infections (HSV). Retrospective data collected from a series of 136 patients demonstrated recurrent HSV in 62% and molluscum infections in 37% of patients [41,138]. More than 90% of affected individuals suffer from recurrent upper and lower tract infections. Some of the pathogens isolated from respiratory tract infections are *Streptococcus pneumoniae, Haemophilus influenzae, Pneumocystis jirovecii*, adenovirus, and respiratory syncytial virus. Abscesses and osteomyelitis by *Staphylococcus aureus* is quite common. Recurrent gastrointestinal tract infections with *Salmonella spp* and *Giardia lamblia* have also been reported [42,43].

### 4.5. STK4 (MST1) Deficiency

This is an autosomal recessive condition characterised by progressive loss of T and B cells due to Fas induced apoptosis. This results from biallelic mutations in serine threonine kinase 4 (*STK4*) also called mammalian sterile 20-like protein 1 (MST1), in the signalling pathway responsible for growth regulation, apoptosis and occurrence of tumours [139]. T and B cell lymphopenia is observed in all patients. Despite reduced B cell counts, serum immunoglobulins including IgE are elevated. Patients suffer from recurrent pulmonary infections with bacteria and viruses [44]. Nehme et al reported 4 patients of Turkish origin with STK4 deficiency. Patients presented within two years of life with recurrent infections caused by *Streptococcus pneumoniae* and *Hemophilus influenzae* which led to bronchiectasis, Varicella zoster virus infections, widespread molluscum contagiosum and persistent Epsein Barr virus infections [45,46]. EBV associated lymphoproliferative disorder has also been described in this deficiency [140].

### 4.6. ZAP70 Deficiency

ZAP 70, a protein tyrosine kinase critical in mediating T cell receptor signaling is deficient in this autosomal recessive condition which affects T cell proliferation and maturation. There is absence of CD8 T cells and inactive CD4 cells. B cells and NK cells are within normal limits [37]. Patients present within two years of life with recurrent severe infections due to lack of functioning T cells. In an instance of hypomorphic mutation late onset of ZAP70 deficiency has also been reported [141]. The condition may be indistinguishable from SCID [37]. In a case report of 3 patients, all had recurrent lower respiratory tract infections, two presented with recurrent oral candidiasis and one patient had disseminated BCG infection [38]. In an eight patient series, three patients had enteritis caused by adenovirus, rotavirus and salmonella [142]. In a screening study carried out among 125 Canadian Mennonites, seven were found to be deficient in ZAP70 and all had oral thrush and otitis media is seen in varying severity [143].

### 4.7. IKAROS Deficiency

Ikaros is a hematopoietic specific zinc finger transcription factor which regulates lymphoid and myeloid differentiation. Loss of function germline mutations in *IKAROS* can result in 2 distinct types of inborn error of immunity diseases: haploinsufficient(HI)mutations presenting as CVID and dominant negative (DN) mutations as combined immunodeficiency [144]. Immunologic abnormalities include almost absent B and NK cells. T cells may be present in normal numbers, but the response to mitogens is absent [145]. Median age of onset is 11 years in haploinsufficient variants. In a cohort of 43 symptomatic patients 34 (79.1%) had where bacterial infections. Respiratory tract infections were the most common with ototis in 23% of patients. *Streptococcus pneumoniae* was the most frequent bacteria isolated. *Streptococcus pneumoniae* has been frequently associated with sepsis and meningitis. Chronic recurrent diarrhoea with *Clostridium difficile, Blastocystis hominis* and *Giardia lamblia* have been reported in 4 patients in this cohort. Other notable infections include sepsis with *Enterococcus gallinarum* and *Hemophilus influenzae.* Viral infections in the form of recurrent herpes labialis by Herpes simplex virus, warts by Human papillomavirus and mumps meningitis are described in 9.3% of patients. Fungal infections are less common in the HI group. Patients with DN alleles suffer from more severe bacterial, viral and fungal infections with a special predilection for *Pneumocystis jirovecii* infection within the first two years of life. Other fungal infections reported are pulmonary aspergillosis, oral candidiasis and *Candida paraspilosis* fungemia. Recurrent viral infections with RSV, adenovirus, influenza, HSV and molluscum contagiosum have been described [40,144].

## 5. Combined Immunodeficiencies with Associated or Syndromic Features

### 5.1. Immunodeficiency with Congenital Thrombocytopenia

#### 5.1.1. Wiskott–Aldrich Syndrome

Wiskott Aldrich Syndrome is an X linked recessive primary immunodeficiency disease resulting from mutations of *WAS* gene. *WAS* gene encodes WAS protein which has a role in regulation of actin polymerization by actin related protein complex [146] The condition is characterised by the classical triad of severe immunodeficiency, eczema and micro thrombocytopenia. T cells are deficient quantitatively and qualitatively. T cells fail to proliferate and secrete IL-2 in response to antigen stimulation. Serum levels of IgG, IgM and IgA are often low with high IgE levels. NK cells may be normal in number but they are functionally defective [147]. Life threatening infections occurred at a median age of 24.8 (range 2–73.9) [148]. In a large cohort of 50 patients, WASP negative patients had 4 times more frequent bacterial infections compared to WASP positive individuals. Severe phenotypes have infections from early infancy. Frequency of infections increase as age advances. The variant and its effect on WAS protein expression determine the degree of immune deficiency [149,150]. In a multinational study where 154 patients were surveyed otitis media (78%), pneumonia (45%) and diarrhea (13%) were the most common infections encountered, but skin infections and systemic infections are also seen. Various viruses like varicella, EBV, CMV, HSV 1 & 2, Polyomavirus and molluscum contagiosum were reported in lesser frequency [88]. Imai et al studied 50 Japanese patients longitudinally and demonstrated that WASP negative are highly susceptible to herpes simplex viral infections. Nine patients in this group had widespread candidiasis. Aspergillosis and PCP were also reported [150].

#### 5.1.2. WIP Deficiency

WAS protein interaction protein (WIP) stabilizes and helps in activating WAS protein which in turn is required for immunological synapse formation, intracellular signalling, cytokine secretion and cellular migration. Deficiency of WIP is an autosomal recessive condition with a combined immunodeficiency picture. Clinical features and laboratory parameters closely resemble Wiskott Aldrich syndrome [89]. T lymphocytes are grossly reduced in numbers. CD8 cells are more affected than CD4.There is an increased proportion of T cell receptor gamma-delta (TCRγδ) positive T cells. B cell numbers are low and their ability to respond to chemokines is diminished. NK cells are also functionally defective. The thrombocytopenia associated with WIP deficiency appears to be less severe and inconsistent [89,90,151]. Patients become symptomatic before 1 year of age. In a mini review by Schwinger et al clinical features of six patients were described. These patients were susceptible to recurrent respiratory infections by CMV and RSV. Chronic diarrhoea and bloody stools were reported in five patients. Rotavirus has been detected in one patient [90,151]. Vesicular skin lesions have yielded *S. epidermidis* and *K. pneumoniae* in the first described case of WIP deficiency [89].

#### 5.1.3. Arp2/3-Mediated Filament Branching Defect

Human Actin related protein 2/3 complex (ARP2/3C) plays a major role in actin filament branching, polymerization and cellular motility. ARP2/3C consists of 7 polypeptides-2 actin related protein subunits (Arp2, Arp3) and 5 regulatory subunits (ARPC1 - ARPC5). ARPC1 exists in 2 isoforms- ARPC1A and ARPC1B. Mutations in *ARPC1B* present with a syndrome of combined immunodeficiency, allergy and autoinflammation which is an autosomal recessive condition [92,152]. Immunophenotyping shows reduced CD4 and CD8 T cells with an increased number of B cells. IgA and IgE are markedly increased. ARPC1B expression is grossly reduced in T, B and NK cells [92,153]. Recurrent upper respiratory tract infections by bacteria and viruses in the form of bronchiolitis and pneumonia are common. Patients are vulnerable to skin infections like abscesses, extensive warts and molluscum contagiosum [91,92]. The first described case of ARP 2/3 C defect had an episode of perichondritis with *Staphylococcus aureus*. He also suffered from chronic bloody diarrhoea and *Salmonella typhimurium* was isolated [152].

## 6. DNA Repair Defects Other than Those Listed in Table 1

### 6.1. Ataxia Telangiectasia (A–T)

This is a complex multisystem disorder described in 1958 is inherited in an autosomal recessive manner characterized by cerebellar ataxia, telangiectasis, immunodeficiency, radiosensitivity and susceptibility to cancers [93,154]. The disease is caused by mutations in ataxia telangiectasia mutated (*ATM*) gene. ATM is a large protein which plays an important role in DNA double strand repair, cell cycle control and survival of cells [155]. B and T cell lymphopenia with impaired lymphocyte functions are seen in a large number of patients. Functional evaluation of T cells have shown in vitro reduced lymphoproliferative responses to antigens leading to cell mediated immunodeficiency. Most common humoral antibody deficiency observed is igG4 deficiency followed by IgA deficiency. A significant number of patients may present with features of hyper IgM [12,155,156]. Some patients may also have normal immunoglobulin levels [93]. Ataxia is generally the presenting syndrome which becomes evident around the time when the child starts to walk. Telangiectasia of exposed sclera is the second major clinical feature followed by susceptibility to recurrent sinopulmonary infections [157]. This can be in the form of recurrent otitis media, sinusitis, recurrent pneumonia which can lead to bronchiectasis and fibrosis of the lung. Common bacteria and viruses are responsible for these infections but persistent fungal or parasitic infections are not generally seen. Majority of patients have *Staphylococcus aureus, Streptococcus pneumoniae,*or *Hemophilus influenzae* as the initial organism isolated from their sputum. Septicaemia due to these bacteria have also been reported. In some of these patients, the respiratory tract later on gets colonized with *Pseudomonas aeruginosa*. Viral agents that cause respiratory infections include respiratory syncytial virus, varicella and Epstein Barr virus. Opportunistic pathogens like *Pneumocystis* *jirovecii* are rarely isolated from the respiratory system of these patients [12,93,94].

### 6.2. Nijmegen Breakage Syndrome

This is a rare autosomal recessive genetic condition grouped under chromosomal instability syndromes. Majority of the cases are from Poland and Czech republic. The gene responsible for Nijmegen breakage syndrome (NBS) has been identified as *NBS1* which codes for the protein named nibrin [97]. The condition is characterised by typical facial appearance, short stature, chromosomal instability and immunodeficiency along with recurrent infections and predisposition to cancers [96]. Immunological findings resemble ataxia telangiectasia. Humoral immunodeficiency due to agammaglobulinemia, deficiencies of IgA, IgG2 and IgG4 are seen. CD3 and CD4 are decreased with inverted CD4/CD8 ratio is consistently reported [96,158]. A predisposition to recurrent respiratory tract infections are seen in all patients. Infections are typically community acquired rather than opportunistic infections. Pneumonia, bronchitis, otitis media, sinusitis and mastoiditis are frequently reported. Urinary tract infections and gastrointestinal infections with diarrhea are seen in 15% of patients [95,96,97].

### 6.3. Bloom Syndrome

Chromosomal instability is the hallmark of this syndrome characterized by short stature with prenatal onset, impaired glucose tolerance and insulin resistance [159], photosensitivity, immune deficiency, infertility and a high risk of developing malignancies, especially leukemias and Non Hodgkin Lymphoma [160]. Lymphocyte subset analysis revealed low T cells with marked reduction of CD4+ cells and naive T cells. Memory B cells were also reduced. Hypogammaglobulinemia may necessitate immunoglobulin replacement [159]. Infections are more common in Bloom’s syndrome when compared to healthy persons but not severe. In a series of six patients, recurrent otitis media and upper respiratory tract infections were common. Bronchitis and pneumonia are also encountered in these patients. One patient in this series had neonatal toxoplasmosis [98].

## 7. Thymic Defects with Additional Congenital Anomalies

### 7.1. CHARGE Syndrome

The CHARGE syndrome is characterized by the occurrence of colobomas, heart defects, choanal atresia, retarded growth and development, genital hypoplasia, ear anomalies/hearing impairment. Although there is considerable overlap with the 22q11.2 syndrome, hypocalcemia and lymphopenia have been found to be more marked. Confirmation of the diagnosis is made by finding a *CHD7* (chromodomain helicase DNA-binding protein) variant [161]. Immunological abnormalities may also include Severe Combined immune Deficiency and humoral defects like hypogammaglobulinemia and IgA deficiency. A presentation with features of Omenn syndrome with autoimmunity has been described [162]. Affected individuals are prone to recurrent upper and lower respiratory infections like otitis media, sinusitis and pneumonia apart from sepsis and septic shock. Recurrent oral thrush is also a troublesome symptom. [101]. In a series of 25 children diagnosed with CHARGE syndrome, 32% died during infancy two of whom died to complications following infection (rhinovirus pneumonia and overwhelming sepsis) [161].

### 7.2. DiGeorge Syndrome

The classic triad of DiGeorge syndrome (DGS) due to 22q11.2 microdeletions consists of conotruncal cardiac anomalies, thymic hypoplasia and hypocalcemia due to hypoplasia of the parathyroid glands. Phenotypic characteristics display a wide and variable spectrum, with cardiac defects being the commonest anomaly, occurring in over 80% of cases. T cell deficiency is the result of thymic hypoplasia and is often mild, resulting in recurrent upper and lower respiratory infections, with no risk of opportunistic or life threatening infections and may reflect the degree of T cell lymphopenia. [163]. Severe T cell deficiency leading to Severe Combined Immune deficiency (complete DGS) occurs in 1% of cases and is incompatible with life without thymic or hematopoietic stem cell transplantation. Newborn screening using TREC assay will pick up complete DiGeorge syndrome [164], which will also be characterized by low CD3 counts and naive T cells with reduced CD45RA expression on CD4+ T cells [165]. Patients usually present with recurrent sinopulmonary infections. Upto one third of patients were found to recurrent sinusitis or otitis media and a minority had pneumonia or bronchitis [166].

## 8. Immuno-Osseous Dysplasias

### 8.1. Cartilage Hair Hypoplasia

This is a rare cause of autosomal recessive rhizomelic dwarfism associated with susceptibility to recurrent or severe infections. Mutations in the ribonuclease mitochondrial RNA-processing *(RMRP)* gene result in abnormalities in multiple systems including defective erythropoiesis, joint hyperextensibility, fine sparse hair, short hands and metaphyseal bony changes. The disease is associated with low T cell numbers and reduced proliferative responses as well as increased numbers of NK cells and carries a higher risk of developing hematological and cutaneous malignancies [167]. Affected individuals are at risk for infections due to a broad range of pathogens including viruses, bacteria and fungi. Recurrent and severe respiratory and gastrointestinal infections are common, resulting in bronchiectasis [103]. Disseminated viral infections due to VZV, HSV, EBV and Parvovirus may occur apart from pneumonia due to *P. jirovecii,* CMV and Aspergillus and oesophagitis due to Candida [102].

### 8.2. Schimke Immuno-Osseous Dysplasia

This multisystem autosomal recessively inherited disorder includes a constellation of defects of the skeletal, haematological, renal, vascular and immunological systems and manifests with short stature and spondyloepiphyseal dysplasia, lymphopenia and frequent infections, progressive renal failure and cerebral ischaemia. Flow cytometry for lymphocyte subset analysis shows low CD3+ T cells with reduced CD4/CD8 ratio and reduced naive T cells. There is an increase in the proportion of CD3+ T cells that show gamma delta TCR, possibly as a result of abnormal T cell differentiation in the thymus [168]. The diagnosis is confirmed by finding a disease - causing variant in the SWI/SNF2-related matrix-associated, actin-dependent regulator of chromatin, subfamily a-like 1 (SMARCAL1) gene. The cause of death is usually sepsis or end stage renal disease, that usually occurs following nephrotic syndrome due to focal glomerulosclerosis [169,170]. Death following CMV pneumonia and encephalitis has also been described [106].

## 9. Hyper IgE Syndromes

### 9.1. Job Syndrome (AD Hyper IgE Syndrome)

Affected individuals have distinctive facial features that usually become recognizable by adolescence, apart from frequent pyogenic and candida infections and eczematous dermatitis.

Recurrent infections of the skin and respiratory tract due to *S. aureus* resulting in boils, furuncles, abscesses, pneumonia with pneumatoceles and empyema occur and cutaneous lesions often lack signs of inflammation, the so called ‘cold abscesses’. Colonization of pneumatoceles with *A. fumigatus* and *P. aeruginosa* may become life threatening due to pulmonary hemorrhage [171] Pulmonary infections due to Non Tuberculous Mycobacteria may occur [172].

Mucocutaneous candidiasis involving the oral and vaginal mucosa and resulting in nail dystrophy is common [29]. Endemic mycoses including meningitis due to *C. neoformans* and *Coccidioides immitis* have been described as also Histoplasmosis causing gastrointestinal infections [108,173,174].

Recurrent trivial trauma fractures, scoliosis, retained primary dentition [175] and increased risk of Non Hodgkin’s lymphoma [176] are also observed. Reduced production of Th17 mediated anti-staphylococcal factors underlies the specific susceptibility to cutaneous and sinopulmonary infections [177] and defective neutrophil chemotaxis has also been described [178]. Serum IgE levels are usually elevated ranging from 1000–50,000 IU/mL [179] and there is striking eosinophilia [180]. Although a positive family history is often obtained, confirmation of the diagnosis requires identification of a pathogenic signal transducer and activator of transcription 3 *(STAT3)* variant.

### 9.2. Comel–Netherton Syndrome

This autosomal recessive disorder of cornification occurs due to mutations in the serine protease inhibitor of Kazal type 5 (SPINK5) gene and results in ichthyosiform erythroderma which often manifests in the neonatal period, a peculiar abnormality of the hair shaft called trichorrhexis invaginata (bamboo hair) and allergic manifestations with elevation of IgE levels [181]. Neonates are prone to develop complications like dehydration, hypothermia and sepsis. Variants in the SPINK5 gene result in defective expression of lymphoepithelial Kazal type inhibitor (LEKTI), resulting in activation of kallikrein (KLK5) and subsequent degradation and chronic inflammation of the epidermal barrier resulting in ichthyosiform desquamating erythroderma. Recurrent bacterial skin infections, conjunctivitis and otitis external also occur apart from bacterial sepsis [110].

## 10. Defects of Vitamin B12 and Folate Metabolism

### Methylene-Tetrahydrofolate Dehydrogenase 1 (MTHFD1) Deficiency

The first patient described with this inborn error of folate metabolism had severe combined immune deficiency and atypical hemolytic uremic syndrome with megaloblastic anemia and elevated homocysteine and methyl malonic acid levels in blood [182].

Microangiopathy, retinopathy, infections, autoimmunity, metabolic acidosis and hepatic fibrosis were described in addition to the features described earlier in patients reported later. Therapeutic response was observed with folic acid and folinic acid, which has been designated as precision therapy to reverse the phenotype. Susceptibility to recurrent pyogenic infections is noted in these patients like pneumococcal septic arthritis, Pneumococcal bacteraemia and periorbital cellulitis [111,183].

## 11. Anhidrotic Ectodermodysplasia with Immunodeficiency

### EDA-ID Due to NEMO/IKBKG Deficiency (Ectodermal Dysplasia, Immune Deficiency)

*IKBKG/NEMO* is essential for activation of the NFkB pathway, which is essential for a plethora of cellular processes involved in immunity, inflammation and proliferation, and mutations result in immune deficiency with ectodermal dysplasia [184]. X-linked hypohidrotic ectodermal dysplasia results from hypomorphic variants in the *IKBKG* gene and affected individuals suffer from recurrent severe infections in addition to the symptoms due to ectodermal dysplasia including the classical triad of hypohidrosis, hypodontia and hypotrichosis. (EDA-ID) Infections of the skin, pneumonia, osteomyelitis, meningitis and colitis occur due to *S. aureus*, *S. pneumonia*, *P. aeruginosa*, *Mycobacteria* and occasionally due to *Pneumocystis*, viruses, or *Candida* [112]. Severe mycobacterial infections due to *M. avium* and *M. kansasii* as well as severe viral infections like encephalitis due to HSV and severe gastroenteritis due to adenovirus infection have occurred [185]. Bronchiectasis is a result of repeated sinopulmonary infections. The immunological derangements include hypogammaglobulinemia, features of hyper IgM syndrome and NK cell dysfunction and the long term outcome is poor without hematopoietic stem cell transplantation, which has been found to result in correction of immune defects with the exception of colitis [186]. EDA-ID with lymphoedema and osteopetrosis has been described [187,188]. Delayed inflammatory responses also are a feature of infections in patients with /EDA – ID [185].

## 12. Calcium Channel Defects

### 12.1. ORAI1 Deficiency

The initiation of signal transduction in various cell types including lymphocytes and Initiation of signal transduction in various cell types including lymphocytes and other cells of the immune system depends on Store Operated Calcium Entry (SOCE), regulated by the ORAI1 and STIM1 genes [114]. Activation of T cell receptors and B cell receptors results in activation of calcium release channels in the plasma membrane, including the well-studied CRAC (calcium release - activating calcium channels), of which ORAI1 is the pore - forming subunit [113].Patients with *ORAI1* deficiency have features of adaptive immune deficiency, congenital myopathy and anhidrotic ectodermal dysplasia with enamel defects of the teeth and dry skin and heat intolerance as a result of anhidrosis. Although lymphocyte subset analysis shows normal numbers of T, B and NK cells, there is significant reduction in T cell activation as demonstrated by poor delayed type hypersensitivity and proliferative responses [189].

### 12.2. STIM1 Deficiency

STIM1, a protein in the membrane of the endoplasmic reticulum activates the ORAI1-CRAC channels by virtue of its role as a sensor of calcium concentrations in the endoplasmic reticulum by multimerization [190] and thus functions as a sensor for calcium influx triggered by depletion of calcium stores [191]. Apart from immune deficiency, myopathy and ectodermal dysplasia observed in ORAI1 deficient patients, STIM1 deficiency has also been found to be associated with autoimmunity [192].Infectious complications include both bacterial infections like sepsis due to *S. pneumoniae* and *E. coli*, otitis media and pneumonia as well as severe viral infections due to CMV, VZV, EBV and enteroviral encephalitis [193].

## 13. Other Defects

### 13.1. Purine Nucleoside Phosphorylase (PNP Deficiency)

This rare autosomal recessively inherited disorder due to mutations in the *PNP* gene is characterized by failure to thrive, progressive neurological abnormalities like developmental delay, spasticity and ataxia along with immunological defects like lymphopenia, T cell deficiency and reduced T cell proliferative responses with normal or reduced B cell function [194]. Autoimmune disorders also occur, especially cytopenias like immune hemolytic anemia [194]. Reduced uric acid levels have also been described [195]. There is enhanced susceptibility to bacterial, fungal, viral and opportunistic infections with onset early in life and respiratory infections including pulmonary tuberculosis with disseminated BCG disease and liver abscesses due to *A. fumigatus* have been reported [115] as well as progressive leukoencephalopathy due to JC virus infection [116].

### 13.2. Immunodeficiency with Multiple Intestinal Atresias

Combined immunodeficiency with multiple atresias involving gastrointestinal tract extending from pylorus to rectum was first described in 1990 [119]. Most cases are sporadic, but autosomal recessive inheritance has also been described. The affected individuals have biallelic mutations in the tetratricopeptide repeat domain 7A (TTC7A) gene [196]. There is loss of architecture, focal scarring and severe inflammation of the gut along with T cell lymphopenia and profound hypogammaglobulinemia and is associated with high mortality [121,122]. Patients suffer from recurrent severe infections by members of Enterobacteriaceae, Pseudomonas spp. and methicillin resistant *Staphylococcus aureus.* Adenoviral infections of respiratory tract and uncontrolled candida infections have also been reported [117,118,119,121,122].

## 14. Combined Immunodeficiency Due to RAG Deficiency

Mild defects including missense variants in the Recombination—activating (RAG) gene may lead to Combined Immunodeficiency with granulomas. [197] Severe viral infections with granuloma formation were observed in patients associated with low T and B cell counts, reduced immunoglobulin levels and thymic size [198]. Late—onset combined immune deficiency with skin granulomas has been described associated with absent B cells, skewed/t cell phenotype and reduced recent thymic emigrants in an adolescent male with compound heterozygous variants in the RAG1 gene [199].

## Figures and Tables

**Table 1:** Microbial spectrum of Infections in IEIs with Combined Immune Deficiency.

Disease—Inheritance	Genetic Defect	Bacterial Infections	Viral Infections	Fungal/Protozoal Infections
CD40 ligand Deficiency XL	*CD40* ligand	*URTI, LRTI—S. pneumoniae, Pseudomonas* [7] Otitis, sinusitis. *Talaromyces marneffei* *S. aureus*, *S. epidermidis*, *E. faecium*, *S. pneumoniae*, *S. maltophilia* and *P. aeruginosa* [8]*TB lymphadenitis* [8]	CMV, EBV and parainfluenza virus [8]	*Pneumocystis jirovecii* [7,8,9,10] *C. parvum* [7,11] *Trichosporon cutaneum* Toxoplasmosis [8]
CD40 AR	*CD40*	Rec pneumonia Septic arthritis [12]		
ICOS AR	*ICOS*	Enteritis Salmonella, Campylobacter Impetigo *S. aureus* Bacteremia *Helicobacter cinaedi* *Sepsis* *E. coli* [13]	Recurrent herpes labialis, herpes keratitis *H. simplex, * Colitis HHV6 Vulvovaginitis, IBD—like symptoms CMV Enteritis Norovirus Adenovirus [13]	Enteritis Cryptosporidium Pneumonia *P. jirovecii* Acute respiratory failure Candida [13]
MHC Class I AR	*TAP1* *TAP2* *TAPBP* *B2M*	Recurrent sinobronchial infections *H. influenzae, S. pneumoniae* *Staph. aureus* *Klebsiella* spp. *E. coli*, *P. aeruginosa* [14,15,16]	Severe viral infections do not occur in isolated MHC class 1 deficiency [16,17,18]	
MHC Class II AR	*CTIIA* *RFXANK* *RFX5* *RFXAP*	Chronic enterocolitis by *Pseudomonas aeruginosa, Escherichia coli* *Salmonella enteritidis* [19,20,21] *Klebsiella pneumoniae, Enterobacter cloacae, Campylobacter jejuni, Proteus mirabilis*, and *P. morgani* [22,23,24,25] *Burkholderia cepacia* [26] *Recurrent pneumonia by* *Streptococcus pneumoniae, Staphylococcus aureus*, and *Haemophilus influenzae* [21,23,27] Adult patient recurrent lung infections *Strep. viridans,* *S.marcensens*, *E.coli (Candida)* *&* Osteomyelitis by *P.mirabilis* and *S.aureus* [28] Bacterial Cholangitis by *Pseudomonas, Enterococcus* and *Streptococcus* spp. [21,23] BCGosis [23,29,30] Meningitis *E. coli* [31]	Meningoencephalitis by Enterovirus, HSV, Adenovirus, live polio virus vaccine [20,21,25,29,31,32] Diffuse interstitial pneumonia by CMV, RSV, enterovirus, adenovirus [20,21,23,32] Hepatitis by CMV [32] Diarrhoea by Rotavirus [22] HPV [33]	Protracted diarrhoea by Candida PCP [22,25,31,32,34,35] *G. lamblia* *C. parvum (sclerosing cholangitis)* [20,21,22,23,36]
ZAP 70 AR	ZAP70	*Severe LRI* [37] Recurrent gastroenteritis, recurrent LRI Recurrent pneumonia BCG osis [38]		*oral candidiasis* [37] Oral thrush [38]
IKAROS AD	*IKZF1*	Severe bacterial infections [39] Recurrent sinopulmonary infections [40] Recurrent sinopulmonary infections, meningiits *S. pneumoniae* Chronic/recurrent diarrhea *C. difficile*, *B. hominis*	Warts HPV Recurrent herpes labialis HSV Mumps Meningitis [39,40]	PCP Pneumonia [39,40]
DOCK8 AR	*DOCK8*	*Recurrent respiratory infections* *Abscesses* [41] *Recurrent otitis externa, Otitis media, sinusitis, mastoiditis, Salmonella enteritis* *Abscesses, osteomyelitis due to S.aureus* *H.influenzae meningitis* [42] Otitis media, mastoiditis, sinusitis, pneumonia, bronchitis. recurrent GI infections *Salmonella enteritis and Giardiasis.* *Sepsis, meningitis* [43]	*Recurrent herpes zoster, molluscum contagiosum* [41] *H. simplex—orolabial, anogenital, keratitis* [42] *Cutaneous viral infections* VZV, HSV, MCV, HPV Keratitis, chronic orolabial, anogenital HSV [43]	*Mucocutaneous candidiasis* [41] *Cryptococcal meningitis* *Nail candidiasis* *Giardiasis* [42] *Pneumonia* *PCP, Histoplasmosis* *Mucocutaneous candidiasis* [43]
STK4 AR	*STK4*	Recurrent upper and lower respiratory infections Recurrent rhinosinusitis Recurrent skin abscesses *Staphylococcal pneumonia* Septicemia [44] *Recurrent skin, lower respiratory infections* *S. pneumoniae, H. influenzae* Recurrent pneumonitis, sinusitis [45]	*Disseminated warts* [44] Recurrent perioral *H. simplex* Extensive *M. contagiosum* Persistent EBV viremia [45] Recurrent Herpes zoster [46]	Mucocutaneous candidiasis [44]
Wiskott Aldrich Syndrome XL	*WAS*	*otitis media, sinusitis, pneumonia, meningitis, sepsis, and colitis* *S. pneumoniae*, *N. meningitidis*, *H. influenzae* [47]	Systemic varicella, CMV infection [48]	*P. jirovecii*, *C. albicans* [48]
WIP Deficiency AR	*WIPF1*	*S. epidermidis* *K. pneumoniae* Vesicular lesions—skin [49]	CMV *pneumonitis* Rotavirus *enteritis* RSV Respiratory distress syndrome [50] Rotavirus Enteritis [49]	
Arp2/3-mediated filament branching defect AR	*ARPC1B*	Recurrent pneumonia, recurrent lymphadenitis, skin abscesses [51] *Bacterial enterocolitis* *Erysipelas* *Skin abscess* *Recurrent otitis media* [52]	*Recurrent viral URI* *Recurrent bronchiolitis*[51] *Extensive warts* *M.contagiosum* *Chronic CMV* [52]	
Ataxia Telangiectasia AR	*ATM*	*Recurrent URI* and *LRI* *Sinusitis, otitis bronchitis, pneumonia* *Sepsis* [53]	Warts, herpes simplex, molluscum contagiosum,, herpes zoster, Uncomplicated varicella, recurrent varicella [54] Varicella pneumonia [55]	Candidal esophagitis Invasive Aspergillosis [55] *P. jirovecii* pneumonia [55]
Nijmegen Breakage syndrome AR	*NBS1*	Pneumonia, bronchitis, otitis media, sinusitis, mastoiditis Urinary tract infections, gastrointestinal infections [56,57,58]		
Bloom syndrome AR	*BLM*	Otitis media Bronchitis Pneumonia [59]		
DiGeorge syndrome AD	Large deletion (3Mb) typically in chromosome 22 *(TBX1)*	Complete DGS Recurrent severe infections, chronic diarrhoea Partial DGS Recurrent sinusitis, otitis, bronchitis, pneumonia [60] Surgical infections Bacterial superinfection after viral infections [61]	*Viral infections* [61]	
CHARGE Syndrome AD	*CHD7* *SEMA3E*	Otitis media, sinusitis, Pneumonia, Conjunctivitis, sepsis *Pseudomonas aeruginosa*, *Stenotrophomonas maltophilia*, *Acinetobacter* Septic shock [62]		Recurrent oral candidiasis
Cartilage Hair Hypoplasia AR	*RMRP*	*otitis media, sinusitis and pneumonia, H. influenzae*, *M. catarrhalis S. pneumoniae* *Sepsis* [63] *Recurrent lung and GI infections* [64]	Severe varicella VZV pneumonia CMV pneumonia Disseminated HSV, EBV, VZV, Parvovirus [63] *Refractory warts, recurrent mucocutaneous HSV infections, severe varicella.* [65]	*Pneumonia* PCP, Aspergillus Thrush [63] *Candida oesophagitis* [65]
Schimke Immuno-osseous Dysplasia AR	*SMARCAL1*	*Sepsis* *Bacterial pneumonia* [66]	*H. zoster* [66] *CMV Pneumonia and encephalitis* [67]	
Job syndrome HIES AD	*STAT3*	*Abscesses, furuncles, cellulitis* *S.aureus* [68] *Recurrent skin abscesses, pneumonia* *Pyopneumothorax, empyema* *S. aureus* *M. abscessus complex* *M. tuberculosis* *Potts spine, abscess* *BCG* *Injection site abscess* [69]		*Skin infections* *C. albicans* [68] *Candidiasis* Oral, nails, lungs, skin Mediastinal mass *A. niger* [69] *Aspergillomas, mycotic aneurysms,* *Colonization of pneumatocoeles* Aspergillus sp *Meningitis, gastrointestinal disease* *C. neoformans* *Endophthalmitis, endocarditis, visceral disease* Candida *Ileocaecal histoplasmosis* *H. capsulatum* [70]
Comel—Netherton Syndrome AR	*SPINK5*	Sepsis [71] *Bacterial skin infections* *Conjunctivitis* *Otitis externa* [72]		
Methylene-tetrahydrofolate dehydrogenase 1 (MTHFD1) deficiency AR	*MTHFD1*		*Varicella* *Influenza* [73]	
EDA-ID due to NEMO /IKBKG deficiency (ectodermal dysplasia, immune deficiency) XL	*IKBG*	*Skin infections, pneumonia, osteomyelitis, arthritis, meningitis, sepsis, colitis* *S. aureus*, *S. pneumonia*, *P. aeruginosa*, *Mycobacteria* [74] *cellulitis, osteomyelitis lymphadenitis, pneumonia, disseminated infections* *M. avium* *M. kansasii* *S. typhimurium* *Klebsiella* *S. marcescens*	*Encephalitis* HSV Gastroenteritis Adenovirus CMV	Pneumonia *P. carinii* Mucocutaneous candidiasis Candida
ORAI1 AR	*ORAI1*	*Pneumonia, Enteritis* *Meningitis* *Pyelonephritis* [75] *BCGitis* *Otitis* *Pyelonephritis* *Meningitis* *Pneumonia* *Chronic diarrhoea* [76]	*Rotavirus enteritis* *CMV infection* [75] *Interstitial pneumonia* *CMV infection* [76]	*Toxoplasma Encephalitis* *Chlamydia pneumonia* [75] *Candidiasis* [76]
STIM1 AR	*STIM1*	*Sepsis* *S. pneumoniae* *E. coli* *Otitis media,* *Pneumonia* [75]	*CMV, VZV* *EBV infection* *Enteroviral encephalitis* [75]	
Purine nucleoside phosphorylase (PNP) deficiency AR	*PNP*	*Recurrent sinopulmonary infections* *lymphadenitis* *Liver abscess* *Disseminated BCG disease* [77]	*Progressive multifocal leukoencephalopathy* *JC virus* [78]	Liver abscess *A. fumigatus* [77]
Immunodeficiency with multiple intestinal atresias AR	*TTC7A*	*Sepsis by Streptococcus faecalis, Pseudomonas aeruginosa* * Enterobacter cloacae, Klebsiella oxytoca Methicillin-Resistant Staphylococcus Staphylococcus hemolyticus, Staphylococcus epidermidis, E faecalis* [79,80,81,82]	*Respiratory infection by Adenovirus* [83]	Uncontrolled Candida infection [84]

## Data Availability

Not applicable.
